# Detection of *magA* Gene in *Klebsiella* *spp*. Isolated from Clinical SamplesDetection* of magA*

**Published:** 2013-02

**Authors:** Alireza Zamani, Rasoul Yousefi Mashouf, Amir Morteza Ebrahimzadeh Namvar, Mohammad Yousef Alikhani

**Affiliations:** 1 Department of Immunology, School of Medicine, Hamadan University of Medical Sciences, Hamadan, Iran; 2 Department of Medical Microbiology, School of Medicine, Hamadan University of Medical Sciences, Hamadan, Iran

**Keywords:** Drug resistance, Iran, *Klebsiella*, Bacterial Proteins

## Abstract

***Objective(s):*** *Klebsiella* infections are caused mainly by *K. pneumoniae* and *K. oxytoca*. In the last two decades, a new type of invasive *Klebsiella pneumoniae* which contains mucoviscosity-associated gene (*mag*A) has emerged. The aim of this study was to investigate the prevalence of *mag*A gene and to detect antimicrobial susceptibility patterns of *Klebsiella* spp. isolated from clinical samples.

***Materials and Methods:*** *Klebsiella* isolates were collected from patients admitted to referral hospitals of Hamadan, Iran, during a 12-month period from 2007 to 2008. The samples were analyzed by conventional microbiological methods and polymerase chain reaction (PCR). The hypermucoviscosity (HV) phenotype of *Klebsiella* isolates was characterized by formation of viscous strings >5 mm as a positive test. The susceptibility of isolates to routine antibiotics was assessed by agar disk diffusion method.

***Results:*** Out of 105 *Klebsiella* isolates, 96.2% was identified as *K. pneumoniae* and 3.8% as *K. oxytoca* by PCR. *mag*A gene was detected in 4 (3.8%) isolates of K. pneumoniae. The isolates of K. oxytoca contained no magA gene. From 4 isolates with positive *mag*A gene, two of them were HV+ and two were HV- phenotype. Overall, sixty-four isolates (60.95%) of *K. pneumoniae* showed an HV positive phenotype and all isolates of *K. oxytoca* were HV- phenotype. The most effective antibiotics against the isolates were tobramycin (79.05%), ceftazidime (79.05%), ceftizoxime (78.09%), ciprofloxacin (76.19%), ceftriaxone (76.24%) and amikacin (74.29%).

***Conclusion:*** The results suggest that there is also magA associated serotype of the *K. pneumoniae* in this region. In addition, the presence of HV+ phenotype may not be associated with *mag*A.

## Introduction

*Klebsiella* infections are caused mainly by *K.* *pneumoniae* and *K. oxytoca*. They are opportunistic bacterial pathogens associated with nosocomial infections such as urinary tract infection (UTI), pneumonia and septicaemia (1, 2). For the first time in 1998, a new type of invasive *K.* *pneumoniae* emerged in Taiwan, which was typically presented as a community-acquired primary liver abscess (3-5). Several reports, especially from the Asia Pacific region and the United States, have also shown that this pathogen has become the predominant cause of liver abscess (6-8). A new virulence gene which is mucoviscosity-associated gene A (*magA*), has recently been identified in this pathogen (9). *magA* is detected in a vast majority of *K.* *pneumoniae* liver abscess isolates and is associated with hypermucoviscosity (HV) and resistance to killing by human serum and phagocytosis (10). 

Based on many recent reports all indicating the emergence of multi-drug resistance *K.* *pneumoniae* (11-14). It seems that the determinantion of antimicrobial susceptibility patterns are essential for appropriate therapy (12). Antibiotic susceptibility pattern of isolates has not been previously determined in our region. Therefore, the aim of this study was to investigate the prevalence of *magA* gene in *Klebsiella* *spp*. isolated from clinical samples and to detect their antimicrobial susceptibility in Hamadan, Iran.

## Materials and Methods

A cross-sectional study was conducted on 105 *Klebsiella* isolates collected from patients. The patients were admitted to referral hospitals in Hamadan, Iran, during a 12-month period from 2007 to 2008. They had no history of antibiotic therapy before sampling and informed consents were obtained from them.The samples were transferred to bacteriology laboratory, plated on MacConkey agar, eosin methylen blue agar, blood agar, incubated for 18–24 hr at 37°C and identified by conventional microbiological methods (15). 

*DNA extraction*

A single colony was taken from each eosin methylen blue agar which had been incubated overnight and emulsified into 100 μl of phosphate buffer salt. After incubation for 10 min at 95°C, 50 μl of proteinase K (100 mg/l) and 150 μl of TE (1 mM EDTA/10 mM Tris, pH 7.5) were added to the suspension and incubated for a further 20 min at 37°C (16). 

For PCR detection, the bacterial DNAs were extracted and amplified using primer pairs targeting specific sequences (Table 1). To identify *K.* *pneumoniae* isolates, 40 pair primers were designed by Oligo software using *urease-D* gene. These primers were tested by Blast software in the web and the best pair was chosen. For detecting *K.oxytoca* and *magA*, specific primers of phenylalanine X (*PehX*) and *magA* genes were chosen from literature (9, 17-19). 

The 20 μl final volume of the PCR mixture contained 2 µl 10x buffer (500 mM-KCl, 100 mM Tris-HCl,pH 8.4, 15 mM MgCl_2_), 0.4, μl of deoxynucleotide mixture (dGTP, dTTP, dATP, and dCTP; 10 mM each), 0.8 μl of MgCl2 (50mM), 1 μl of each primer (10 mM), 0.1 μl Taq polymerase (5 units), 1 μl of template DNA and 13,7 µl distilled water.For amplifying condition, the initial denaturation step of 2 min at 94°C was followed by 35 cycles of 45 s at 94°C, 45 s at 60°C for *ure-D*, 59°C for *PehX* and 52°C for *magA*, 45 s at 72°C and the extension step of 5 min at 72°C. PCR products were detected by electrophoresis on 1% agarose gel. Finally, for *magA*, the related bond was prepared by DNA extraction kit from gel and was sequenced (Milegen, France) (20).

The hypermucoviscosity (HV) phenotype of *Klebsiella* isolates was also characterized and formation of viscous strings >5 mm in length showed a positive string test or a mucoviscose shape when a loop was passed through a colony (17). 

In order to detect susceptibility of isolates to routine antibiotics, all isolates of *K.* *pneumoniae* and *K. oxytoca* were assessed by an agar disk diffusion method recommended by Clinical and Laboratory Standard Institute (21). Ten antibiotics including tobramycin (10 µg), amikacin (30 µg), gentamicin (10 µg), ceftriaxone (30 µg), ceftizoxime (30 µg), cefalotine (30 µg), cefatazidime (30 µg), cefazolin (30 µg), nalidixic acid (30 µg), and ciprofloxacin (5 µg) were used for the antibiogram. *K.* *pneumoniae* ATCC 1290, *K.oxytoca* ATCC 1402 and *E. coli* ATCC 11303 were used as reference strains (20). 

## Results

Out of 105 *Klebsiella* isolates, 38.1% were isolated from urine, 30.4% from stool, 11.4% from liver abscess, 4.8% from blood, 3.8% from wound, 3.8% from sinus, 2.9% from sputum and 4.8% from unknown samples. From 105 *Klebsiella* isolates, 92 strains (87.6%) were identified as *K.pneumoniae*, 5 strains (4.7%) as *K. oxytoca* and 8 strains (7.6%) as *Klebsiella* *spp*. by conventional microbiological tests, compared to 101 strains (96.2%) as *K.* *pneumoniae* and 4 strains (3.8%) as *K. oxytoca* by PCR. 

*MagA* gene was detected in 4 isolates (3.8%) of *K.* *pneumoniae*, but none of the isolates of *K. oxytoca* contained *magA* gene. From the 4 isolates, three (75%) were obtained from blood samples and one (25%) from an abscess sample. Sixty-four isolates (60.95%) of *K.* *pneumoniae* showed an HV positive phenotype. From 4 isolates with positive *magA* gene, two of them were HV^+ ^and two were HV^-^ phenotype. The 4 isolates (100%) of *K. oxytoca* were HV^-^ phenotype.

The results of the susceptibility of 105 isolates of *K.* *pneumoniae* and *K. oxytoca* to ten routine antibiotics are shown in the Table 2.

**Table 1:** Details of specific oligonucleotides which were used as primers to amplify particular sequences of Klebsiela pneumonia, K. *oxytoca* and *magA* gene

Gene	primers	Size of amplicon	GenBank Accession no.	Ref.
*Ure-D*	5'-CCC GTT TTA CCC GGA AGA AG-3'	243 bp	L07039	This study
	5'-GGA AAG AAG ATG GCA TCC TGC-3'			
*PehX*	5'-GAT ACG GAG TAT GCC TTT ACG GTG-3'	344 bp	AYO65648	(19)
	5-TAG CCT TTA TCA AGC GGA TAC TGG-3			
*magA*	5'-CGC CGC AAA TAC GAG AAG TG-3'	540 bp	AB085741	(9)
	5'-GCA ATC GAA GTG AAG AGT GC -3'			

**Table 2:** Susceptibility of 105 isolates of Klebsiela *pneumoniae* and K. *oxytoca* to ten routine antibiotics

Antibiotic	Sensitive No (%)	Resistant No (%)	Antibiotic	Sensitive (%)	Resistant (%)
Tobramycin	83 (79.05)	22 (20.95)	Amikacin	78 (74.290	27 (25.71)
Ceftazidime	83 (79.05)	22 (20.95)	Gentamicin	75 (71.430)	30 (28.57)
Ceftizoxime	82 (78.09)	23 (21.90)	Nalidixic acid	75 (71.430)	30 (28.57)
Ciprofloxacin	80 (76.19)	25 (23.81)	Cefalotine	65 (61.91)	40 (38.09)
Ceftriaxone	79 (75.24)	26 (24.76)	Cefazolin	64 (60.95)	41 (39.05)

Tobramycin (10 µg), Ceftazidime (30 µg), Ceftizoxime (30 µg), Ciprofloxacin (5 µg), Ceftriaxone (30 µg), Amikacin (30 µg), Gentamicin (10 µg), Nalidixic acid (30 µg), Cefalotine (30 µg) and Cefazolin (30 µg

**Figure 1:**
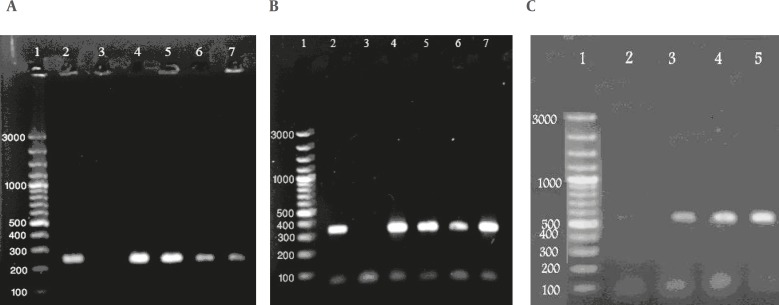
Agarose gel electrophoresis analysis for the Ure-D gene in Klebsiela *pneumoniae* (A), the *PehX gene* in K. *oxytoca* (B) and *magA* gene in K. *pneumonia* (C) strains isolates

**Lane 1, molecular size marker, expressed in base pairs. Lanes 2A,** ***K.*** ***pneumoniae* (ATCC 1290), 243 bp and 2B, *K. oxytoca*e (ATCC 1402), 344 bp as positive controls. Lanes 3A, 3B and 2C, Escherichia coli (ATCC 11303) as negative controls and the other wells as positive isolates**

## Discussion

The purpose of the study was to investigate the presence of *magA* gene in *K.* *pneumoniae* and *K. oxytoca*, which were isolated from clinical samples. In Fang’s study, 52 out of 53 (98 %) *K.* *pneumoniae* isolated from liver abscess carried this specific virulence gene and the presence of one *magA*-negative isolate was thought to be due to patient’s underlying disease of liver cirrhosis and hepatic failure. Thus, Fang *et al.* concluded that *magA* is an essential virulence gene for *K.* *pneumoniae* strains causing liver abscess and could be used as a diagnostic tool. Fang believed that *magA* gene is exclusivly limited to liver abscess and HV positive phenotype (9, 10, 22). With extension of global research in other countries such as North American countries, Singapore and Korea, on *Klebsiella* isolates, they showed *magA* gene isolated from other cases like acquired bacteraemia, sepsis, meningitis and endophthalmitis (5, 6, 8, 17, 23, 24). In contrast to Fang’s studies, these samples included HV^+^ and HV^-^ phenotypes. Therefore, based on the results of the present as well as other studies, containing HV^+ ^phenotype is not a certain reason for the presence of *magA* gene since the HV^-^ phenotype may have *magA* gene, too (17, 25). In our study, we found 4 positive *magA* gene isolates while two of them contained HV^+ ^and two were HV^-^ phenotype. Based on the data of this project, *magA* gene can specially belong to *K.* *pneumoniae* because we examined the *K. oxytoca* isolates for the presence of *magA* gene but none of them carry this gene.

The survey of antibiotic susceptibility of *K.* *pneumoniae* and *K. oxytoca* showed different percentages of susceptibility to the tested antibiotics. In this study resistance to nalidixic acid, cefalotine and cefazolin was relatively high in contrast to other studies (13, 14, 26). In our study, tobramycin was mostly active against strains of *Klebsiella* followed by ceftazidime and ceftizoxime.

## Conclusion

The *K.* *pneumoniae* *magA* positive strains exist in our area and are isolated from various samples. In addition, the presence of HV^+ ^phenotype is not associated with *magA* gene. 
